# The Role of Transcranial Ultrasound Imaging in Intensive Care Treatment of Decompressive Hemicraniectomy Patients: A Retrospective Single-Center Analysis

**DOI:** 10.3390/jcm13247704

**Published:** 2024-12-17

**Authors:** Martin Petkov, Ralf Becker, Max Schneider, Michal Hlavac, Andreas Knoll, Christian Rainer Wirtz, Ralph König, Andrej Pala

**Affiliations:** 1 Department of Neurosurgery, Bezirkskrankenhaus Günzburg, University of Ulm, Lindenallee 2, 89312 Günzburg, Germany; 2 Department of Neurosurgery, University of Ulm, Albert-Einstein-Allee 23, 89081 Ulm, Germany; 3 Department of Radiology, University of Ulm, Albert-Einstein-Allee 23, 89081 Ulm, Germany

**Keywords:** transcranial ultrasound, hemicraniectomy, intensive care unit, bedside

## Abstract

**Background**: Post-hemicraniectomy patients often need extended intensive care treatment. While computed tomography (CT) is considered the gold standard for regular imaging, its frequent use could be linked to adverse clinical outcomes. This study aimed to assess bedside transcranial ultrasound (TUS) to capture intracranial anatomical structures and pathologies. **Methods**: We analyzed 19 patients treated in our neurosurgical ICU from 1 January 2023 to 1 February 2024. Six physicians from our unit (three residents and three attending physicians) conducted a retrospective evaluation. A total of 158 sessions, including multiple freeze frames and video footage, were analyzed, including 7 imaging categories, using a Likert scale. Subsequently, correlation between CT and TUS was evaluated for midline (ML) shift, subdural space, lateral ventricular width (LVW), and extent of intracerebral hematoma using the Pearson’s correlation coefficient (r). **Results**: TUS was performed on average on 8.32/19.53 days (mean inpatient stay). It provided the lowest Likert scores for the imaging categories ventricular system, midline, subdural space, intraventricular catheter placement, and cortical gyration. Residents reported slightly inferior assessability, resulting in higher scores on the Likert scale (0.02–0.93 mean difference compared with attending physicians). A high correlation was shown in terms of ML shift, LVW, and intracerebral hematomas. No relevant correlation was shown in subdural space. **Conclusions**: TUS is a safe, cost-, and time-efficient method, potentially gaining relevance for imaging post-hemicraniectomy patients. In our setting, the method seemed effective in depicting intraventricular catheter placement, hydrocephalus, ML shift, and space-occupying lesions. Further improvement in image quality could potentially reduce the overall number of indicated CT scans.

## 1. Introduction

Decompressive hemicraniectomy is a standard neurosurgical procedure aimed at reducing intracranial pressure in patients following traumatic brain injuries, intracranial hemorrhages, and extensive cerebral insults [1]. Affected patients often require long intensive care treatment and show prolonged dependency on mechanical ventilation [2,3]. Aside from adequate intracranial pressure management, regular imaging checks are crucial for the assessment of compromised anatomical structures, early detection of hydrocephalus, postoperative complications and determination of further surgical therapy [4,5]. In terms of the rapid morphological assessment of parenchymal structures, computed tomography (CT) is considered the gold standard for primary imaging within intensive care unit (ICU) treatment [6]. This necessitates multiple transfers and transportation of the patients, which may lead to increased intracranial pressure during manipulations like head movement and flat positioning on the CT table [7,8].

Several studies have reported a potential negative impact of repetitive CT scans on critically ill patients within the neurosurgical treatment [6,9,10]. Transcranial ultrasound (TUS) is considered a radiation-neutral, inexpensive, and portable diagnostic tool for assessing intracranial abnormalities in young children before fontanelle closure [7]. In 1982, Rune Aaslid described a non-invasive method for transcranial Doppler (TCD) sonographic assessment of blood flow velocity in the basal cerebral arteries [11]. In the following years, Albrecht Harders and colleagues refined the technique into a clinically practical, atraumatic bedside procedure for measuring blood flow velocity in the major arteries of the Circle of Willis [12,13]. TUS also gained importance in neurosurgery within the operating room. In the early 1980s, Dohrmann and Rubin used intraoperative ultrasound to examine several patients with intracranial masses, successfully visualizing a variety of different lesions [14]. Over time, this technique evolved further, enabling real-time tumor localization and precise delineation of resection margins. With ongoing technological advancements and improved resolution, intraoperative ultrasound has become a sensitive and valuable tool in tumor surgery [15]. Outside the operating room, TUS is routinely utilized in pediatric neurosurgery, where it is widely used through the fontanelles to evaluate intracranial injuries following trauma or to assess conditions such as hydrocephalus [16,17].

In adult patients, a morphologic evaluation often is not possible due to bone structures and associated shadow formations. In the case of post-hemicraniectomy patients, absence of the skull can allow the depiction of deeper structures of the brain [18].

However, there are limited studies in humans regarding the optimal usage and indication of TUS in bedside intensive care treatment. The aim of this study was to evaluate the quality of this frequently used imaging tool for assessing various structures and intracranial processes and to evaluate the comparability to CT within the neurosurgical ICU treatment.

## 2. Materials and Methods

A total of 19 post-hemicraniectomy patients treated in our neurosurgical ICU and examined by TUS between 1 January 2023 and 30 June 2024 were analyzed. Inclusion criteria were age over 18 years and the absence of a wound infection. A CT scan was performed as baseline assessment no later than 48 h after the initial surgery. Demographic data such as age and gender were collected for the patient cohort, along with primary diagnosis, length of hospital stay, and the number of CT scans per patient. Imaging of the cerebral morphology was conducted transtemporally by a Canon Aplio A device with a curved 8.1 MHz array through the craniectomy defect. Six physicians from our neurosurgical ICU (three residents and three attending physicians) conducted a retrospective evaluation of the digitally saved recordings.

A total of 158 sessions, including multiple freeze frames and video footage, were analyzed in seven imaging categories: ventricular system, midline (ML), subdural space, intraventricular catheter placement, cortical gyration, intracerebral hematoma (ICH) and infarct demarcation area (if present). Ratings were performed using a Likert scale (1 point: very good visualization; 5 points: insufficient visualization; 6 points: structure not found). For each imaging category, mean values and standard deviations were calculated for all six examiners and separately for residents/attending physicians. Both groups were compared with each other. 

For quantitative assessment, the ML shift, width of the subdural space, and the lateral ventricular width (LVW), as well as the maximum measurable diameter of ICHs, were correlated between CT and TUS. Data with a time interval less than 24 h between applications of both imaging modalities were included. Subsequent surgical revisions, VP-shunt implantations, or changes in the cerebrospinal fluid (CSF) drainage regimen (e.g., adjustments of external ventricular drainage (EVD) height) resulted in the exclusion of the corresponding images. A total of 16/19 patients, 40/158 TUS sessions, and 271 sequences (freeze frames and video footage) matched the inclusion criteria. ML shift in TUS was determined according to the protocol of Maramattom et al. by measuring the distance from the ipsilateral duraplasty to the center of the third ventricle (A) and the distance from the center of the third ventricle to the contralateral inner bone (B). The difference between both measurements (B–A) was divided by two [17]. For evaluation in CT, the distance between the ideal midline (between anterior and posterior falx) and the septum pellucidum was measured where the greatest deviation was observed (according to the American Association of Neurological Surgeons) [18]. As a parameter of the Evans Index, lateral ventricular width was correlated by measuring the maximum axial distance between the frontal horns in both imaging modalities [19]. Subdural space and ICHs (if present) were measured by the maximum width or diameter, respectively, that could be measured in each imaging modality. For the subdural space, the maximum distance between the dura or duraplasty and the cortex was measured. 

The linear relationship between both produced datasets was correlated by calculating a Pearson’s correlation coefficient.

## 3. Results

The primary underlying conditions during our intensive care treatment were spontaneous subarachnoid or intracerebral hemorrhages as well as large infarcts with malignant edema, necessitating urgent decompressive hemicraniectomy.

A typical TUS session included multiple freeze images and video recordings. The latter had a duration of up to 30 s and consisted of several hundred image sequences. The demographic and clinical data of the patient collective are summarized in Table 1.

### 3.1. Imaging and Evaluation of Anatomical Landmarks

**Figure 1:**
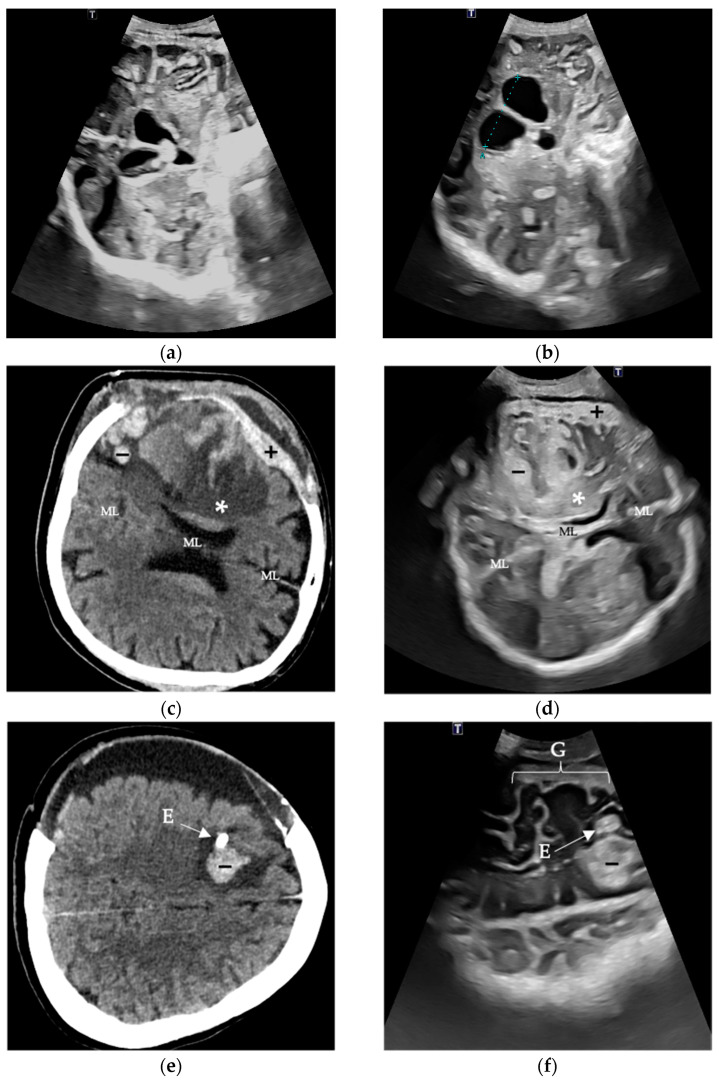
Evaluation of predefined image markers in selected cases In the first case, the coronal view of the lateral ventricles and the third ventricle with opened EVD (**a**) and progression in ventricular growth after 24 h EVD closure (**b**) is shown. In the case of a hemorrhagic infarction in a 65-year-old patient, both the corresponding cranial CT (**c**) and the follow-up TUS examination (**d**) reveal infarcted brain tissue (*), a subtle subdural hemorrhage (+), as well as intracerebral hemorrhagic components (−). A relevant midline (ML) shift is not present. Finally, a cranial CT (**e**) and TUS (**f**) of a 41-year-old patient with spontaneous SAH is depicted. In this case, in order of EVD (E) implantation, a consecutive tract hemorrhage occurred that was evaluated daily. Also, gyration (G) with corresponding external cerebrospinal fluid spaces could be assessed via TUS in the absence of postoperative hemorrhage.

A collection of selected patient examples for evaluation of the predefined image markers is shown in Figure 1.

**Figure 2:**
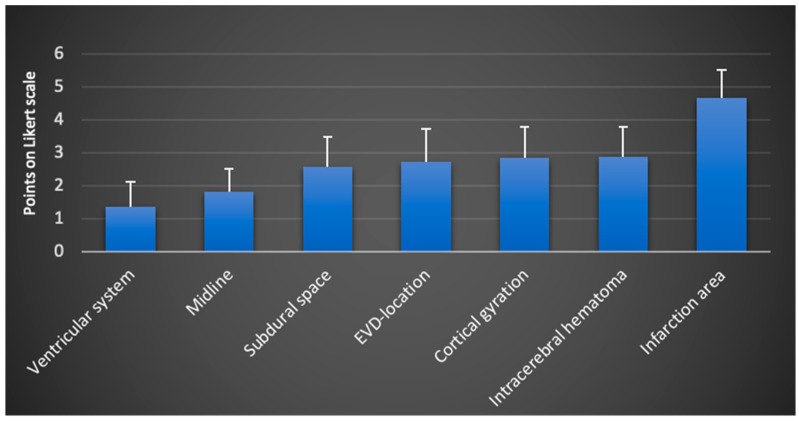
Total scores including all examiners (mean values and standard deviation on the Likert scale from 1: very good visualization to 5: insufficient visualization and 6 points: structure not found; N = 6 examiners; n = 158 TUS sessions).

In most cases, multiple anatomical and pathological image markers could be assessed from a single image. In some instances, a focused examination was conducted when addressing a specific clinical question. For the qualitative evaluation, all available images and videos from the examinations were utilized. The overall results of the evaluation are presented in Figure 2.

Overall, the image markers ventricular system, ML, and subdural space received the best (lowest) scores. Regarding the EVD location, all examiners noted a negative impact from a tract hemorrhage, which occurred in two cases (see Figure 1f). ICHs were correctly identified by all examiners, although the presence of a resorption edema or infarct in close proximity negatively affected assessability. By a large margin, the visualization of infarction areas received the lowest ratings from all examiners. 

### 3.2. Evaluation and Comparison of the Resident and Attending Subgroup

**Figure 3:**
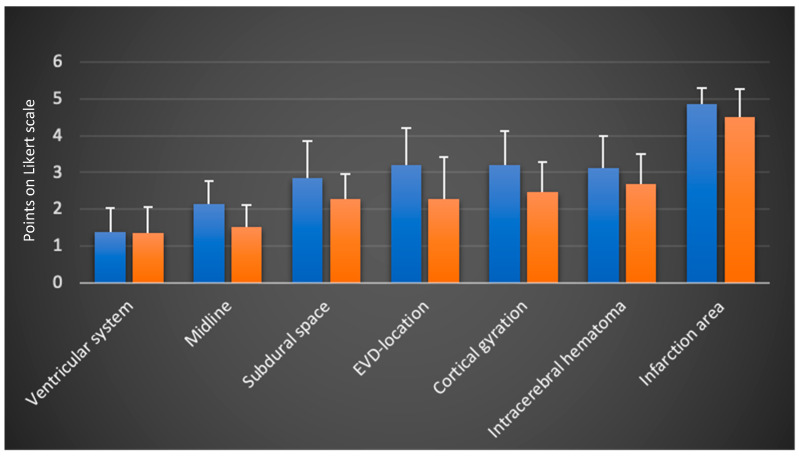
Separated scores of residents (left column) and attending physicians (right column) (mean values and standard deviation on the Likert scale from 1: very good visualization to 5: insufficient visualization and 6 points: structure not found; N = 3 examiners; n = 158 TUS sessions).

A separate analysis and comparison of the two subgroups—residents and attending physicians—was conducted (Figure 3). In the separate evaluation, the more experienced attending physicians gave lower (better) ratings in all categories.

The ranking trends among the imaging categories remained consistent. The mean ratings and differences between both groups are summarized in Table 2. The largest differences between the two subgroups were observed in the categories EVD location, cortical gyration and midline.

### 3.3. Quantitative Correlation of TUS and CT

**Figure 4:**
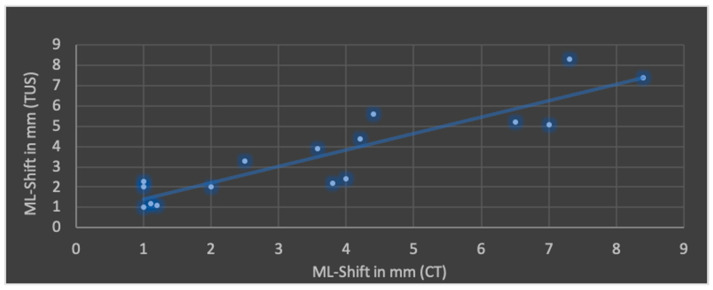
Correlation of transcranial ultrasound (TUS) and computed tomography (CT) in terms of midline (ML) shift (r = 0.90; *p* < 0.001; mean value TUS: 3.6 ± 2.2 mm; mean value CT: 3.7 ± 2.5 mm).

**Figure 5:**
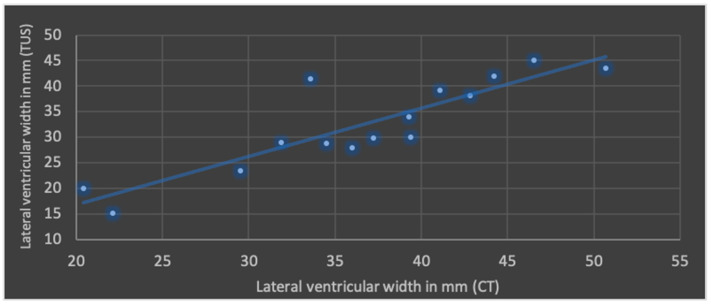
Correlation of transcranial ultrasound (TUS) and computed tomography (CT) in terms of lateral ventricular width (r = 0.88; *p* < 0.001; mean value TUS: 32.5 ± 9.0 mm; mean value CT: 36.6 ± 8.4 mm).

**Figure 6:**
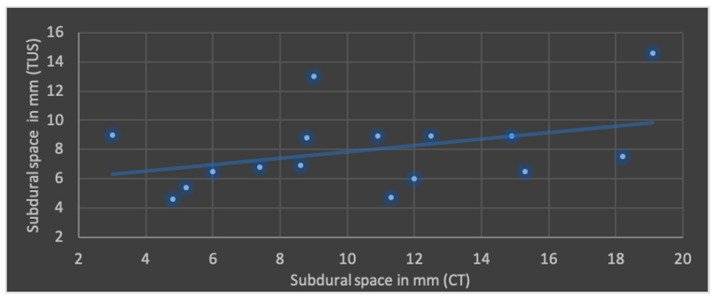
Correlation of transcranial ultrasound (TUS) and computed tomography (CT) in terms of subdural space (r = 0.39; *p* = 0.14; mean value TUS: 7.9 ± 2.7 mm; mean value CT: 10.4 ± 4.7 mm).

**Figure 7:**
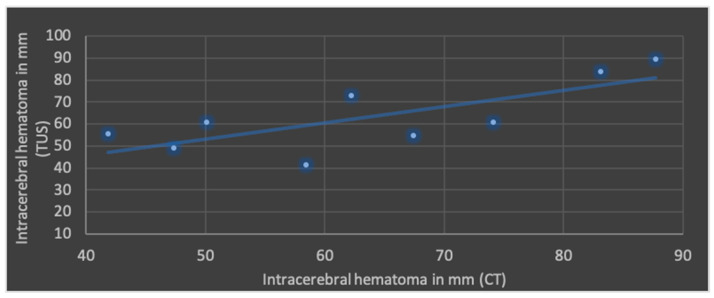
Correlation of transcranial ultrasound (TUS) and computed tomography (CT) in terms of intracerebral hematomas (r = 0.74; *p* < 0.05; mean value TUS: 63.3 ± 15.9 mm; mean value CT: 63.6 ± 16.0 mm).

The Pearson’s correlation coefficient (r), including mean values and standard deviation of both modalities in terms of ML shift, lateral ventricular width, subdural space and ICH is shown in Figure 4, Figure 5, Figure 6 and Figure 7.

The best correlation represented by Pearson’s correlation coefficient was shown in ML shift and lateral ventricular width. The evaluation of ICHs in TUS revealed a slightly weaker correlation, but with a *p*-value below 0.05, still indicating significance at the standard level. No significant correlation was found in terms of subdural space between both modalities; mean values obtained through CT were higher than those in TUS.

### 3.4. Patient Outcome and Complications

Prior to the indication for hemicraniectomy, 17 out of 19 patients (89.5%) were intubated, sedated, and mechanically ventilated. Using TUS, surgery-associated complications requiring re-intervention were identified in 4 of 19 patients (21.1%): 2 epidural hemorrhages and 1 intracerebral hemorrhage after hematoma evacuation, all requiring surgical revision. In one case, a growing cerebrospinal fluid collection was punctured under ultrasound guidance, avoiding further surgery. In 4 out of 6 patients with hydrocephalus following EVD closure and the need for VP shunt implantation, the indication for surgery could be determined without the need for additional CT imaging (Figure 1a,b). In two cases of detected tract hemorrhages following EVD placement (one case shown in Figure 1e,f), neither required surgical revision. Three out of nineteen patients (15.8%) were palliated during their hospital stay due to a poor preexisting medical condition and uncontrollable infection with unfavorable prognosis. Eight out of nineteen patients (42.1%) remained dependent on additional ventilatory support after tracheotomy until transfer to the early rehabilitation facility. In patients requiring re-surgery, an average of 15 TUS sessions and 8 CT scans per patient and stay were performed. 

## 4. Discussion

Decompressive hemicraniectomy is performed to rapidly reduce increased intracranial pressure due to traumatic brain injuries, intracranial hemorrhages, as well as space-occupying brain infarcts. Affected patients often require extended intensive medical therapy and have impaired transport capability. The gold standard for imaging to assess treatment, prognosis, and complications is CT, typically performed in stationary settings. However, it has several drawbacks, including radiation exposure, limited availability at the bedside, less repeatability and high cost. Frequent repositioning and head manipulation can also negatively impact intracranial pressure, which could be associated with poor clinical outcomes, highlighting the need for alternative imaging methods. In the present study, bedside TUS was performed on 19 post-hemicraniectomy patients of our neurosurgical ICU and retrospectively evaluated in terms of imaging quality and assessing various structures and pathological processes.

### 4.1. Technical Background

Diagnostic ultrasound uses a piezoelectric transducer to generate high-frequency sound waves, which are transmitted into tissue and reflected back as echoes based on the tissue’s acoustic properties. These echoes are converted into electrical signals and processed into a gray-scale, 2D image, with brightness determined by the amplitude and timing of the echoes [20]. Transcranial sonography for imaging intracranial structures in adults is considered a challenging modality due to the fact that ultrasound waves are strongly reflected by bone, which hinders the adequate visualization of underlying structures such as the brain [21]. For morphological imaging in cranial neurosurgery, ultrasound is primarily used intraoperatively after the bone has been removed or in areas where the skull is thinner, such as the temporal bone. However, even in these regions, it is difficult to assess brain anatomy clearly [6]. While the correct use of ultrasound gel is essential for adequate transmission, the selection of the transducer is crucial for successful imaging. It must offer both high resolution and adequate tissue penetration to allow clear visualization of intracranial structures [22,23]. In our setting, a curved 8.1 MHz array was chosen, combining a wide representation and sufficient penetration of deeper cerebral structures.

Different intracranial structures can exhibit varying echogenicity: gyri are mostly isoechogenic; cerebrospinal fluid (CSF) is hypoechogenic; and structures like the falx, sulci, and dura mater typically are hyperechogenic [24]. Unlike CT, ultrasound echogenicity, in some cases, can be angle-dependent (e.g., connective tissue), which can impact the assessment [25]. This can also be affected by pathologies with structural changes over time. Acute ICHs appear hyperechogenic, while older post-hemorrhagic changes often appear as hypoechogenic lesions [24]. During the resorption phase, varying echogenicities can be observed (as shown in Figure 1d,f).

### 4.2. Literature Review and Interpretation of the Study Results 

Regarding the application of TUS in post-craniectomy patients, the literature predominantly consists of case studies and studies with small patient cohorts [1,5,18,26]. Only a few studies compare CT and TUS in terms of visualizing relevant structures, and they are mostly obtained through the temporal acoustic window in non-craniectomized patients [5,27,28,29]. In a recently published prospective pilot study by Chouhan et al., 40 post-hemicraniectomy patients were examined. A strong correlation between both imaging modalities was observed in terms of ventricular width. However, a high standard deviation for ultrasound measurements was noted in a few patients with a severely enlarged ventricular system. Identification of hyperechoic lesions was described as more effective than hypoechoic ones. A correlation of lesion size (diameter or volume) was not performed. The working group describes the curved transducer as the most useful for this purpose. This corresponds with our study, where a curved probe was also selected [24]. In an older study by Bendella et al., 102 patients were examined using TUS. Ultrasound was performed no later than 24 h after cranial CT, which corresponds with our time frame. A strong correlation was found for ventricular diameters and ML shift. In this analysis, as in our study, the Pearson correlation coefficient was determined. Regarding lateral ventricular width and ML shift, slightly higher coefficients were observed here, although both study results are statistically significant. It cannot be ruled out that the prospective design and standardized study protocol as well as the larger patient number contributed to this outcome. TUS examinations were performed by three experienced investigators, analogous to our study. A qualitative assessment of the examiner subgroups was not conducted [6].

Another study by an anesthesiologic department included 30 head-injured patients treated in the ICU. In addition to similar findings regarding ML shift and ventricular width, they also found a strong correlation between the volumes of hyperdense lesions in CT and TUS. However, ischemic areas could not be adequately visualized and there was poor correlation in the volume of late hemorrhagic lesions [30]. In our study, the depiction of infarcted areas via TUS proved to be challenging. Smaller infarct areas were not always identified (six points on the Likert scale). Similarly, the qualitative evaluation of older hemorrhages showed relatively poor results. Although intracerebral hematomas could be identified, all six investigators considered it demanding to determine the extent and exact boundary of the lesion compared with healthy tissue (especially during the resorption phase and decreasing echogenicity). Interestingly, when comparing the qualitative results of the Likert scale with our quantitative analysis of ICHs, partially contradictory findings emerge. In contrast to the subjectively challenging identification of the extent of lesions, a strong correlation of the maximum diameter measured in TUS and CT was determined. This highlights the need to distinguish between two aspects: identifying the complete boundary of the lesion from healthy brain tissue—which appeared particularly challenging in TUS in our study, especially in cases of resorption edema—and estimating the approximate size of the mass effect itself. It is also noteworthy that the maximum diameters identified in our study suggest larger intracerebral hematomas compared with those reported by Caricato et al., which may also influence comparability [6,30].

To our knowledge, no large study has been published that qualitatively assesses EVD placement and localization by TUS in post-hemicraniectomy patients. It is known that sonography can be used during EVD placement in these patients [31]. In our case, the presence of a hematoma adjacent to or surrounding the EVD was associated with higher scores on the Likert scale and a more difficult evaluation of the catheter trajectory.

Overall, the timeframe of our study between TUS and CT imaging is situated in the upper range compared with existing works (Table 3). Nevertheless, the qualitatively and quantitatively determined parameters above are comparable with the mentioned studies. In cases of ICHs and subdural space, comparable studies are lacking. This is particularly relevant since postoperative re-hemorrhages in various compartments of the surgical site are among the most common complications following a craniectomy, making an evaluation of the applicability of TUS for assessing re-bleeding important [32]. In cases of subdural hematomas, no significant correlation was observed in our study. One reason for this could be the pressure exerted on the defect and redistribution of fluid, resulting in a smaller width. Depending on the positioning of the patient, the subdural space also can become smaller, especially with an elevated upper body position, which can influence comparability negatively.

### 4.3. Technical Limitation and Pitfalls

Although the curved transducer is used routinely in several studies mentioned above, especially when depicting structures through the craniectomy defect, one disadvantage of using the curved transducer is that, particularly in cases of large defects (e.g., large CSF collections), the contact surface may be reduced, requiring increased pressure. This pressure may compress the skin, potentially redistributing underlying fluid and leading to its underestimation. An example of this can be seen in Figure 1e,f, where subdural and epidural CSF collections are evident on CT but only minimally visible on TUS. A relatively high examiner dependency of ultrasound in general is well known [33]. 

The performance of TUS as well as sonography in general is often less standardized compared with a predefined CT protocol, which can be another potential pitfall in receiving valid results [16,34].

In addition to the potential transmission of infectious agents through repeated repositioning of the patient and transport to the CT, ultrasound can also pose an infection risk if handled improperly. Regular disinfection, not only of the transducer but also of the device itself, is essential, particularly when in contact with patients in protective isolation [35]. Ultrasound gel, in particular, has been associated with infection outbreaks in various settings worldwide, and the risk of contamination from non-sterile ultrasound gel has been documented [36,37,38]. In cases of existing superficial wound infections, excessive use of ultrasound was avoided in our setting. The use of sterile gel, along with alternative antiseptic agents, could potentially reduce this risk. However, applying pressure to the not-fully-healed hemicraniectomy scar could still entail the risk of infection [39]. 

### 4.4. Proposal of Time Schedule and Suggestions

Established guidelines or standardized time schedules for bedside post-(hemi)craniectomy TUS in the ICU have not been published yet. Based on our experiences, the vulnerable patient cohort and the available data, we recommend the following routine when applying TUS in this patient population: After the first postoperative CT, a correlating TUS should be performed as soon as possible. It is essential to maintain consistent patient positioning while performing TUS, which should be continued throughout the later course of the hospital stay. A horizontal patient position is not recommended in the case of an ICP crisis [6]. A supine position with the upper body elevated at 30° has proven effective in our practice. The sequences should be performed in a predefined position over the defect, as previous prospective studies have shown [6,30]. Before the first TUS, the optimal position should be documented in accordance with the best achieved image quality and anatomical landmarks. These could be the distance from the lateral canthus to the external auditory canal or predefined marks like the orbitomeatal line, according to Kern et al. [40,41]. For comparability of the recordings, it is optimal to perform video sequences with the transducer positioned both horizontally (for axial scan) and vertically (for coronal scan) and at minimal and maximal achievable angles (by angling over the defect) to depict all relevant structures [24]. A focused ultrasound for specific clinical questions can be performed independently of the protocol. Knowing the existing interobserver variability of TUS in infants and TCD, regular training of the examiners by experienced colleagues is essential to reduce bias in imaging results and prevent incorrect therapeutic conclusions [16,42]. 

### 4.5. Selected Cases and Special Indications for TUS

**Figure 8:**
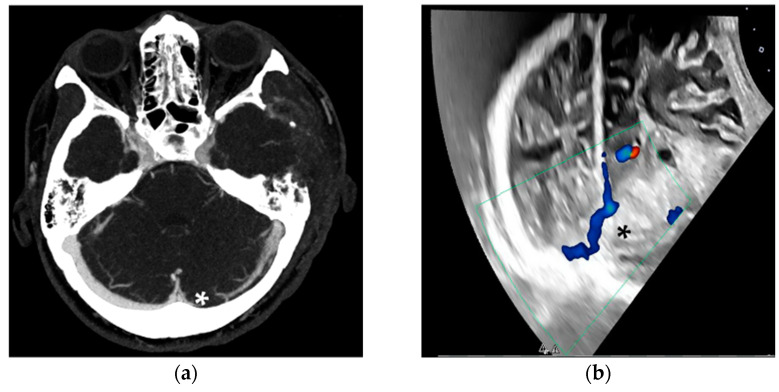
Venous CT angiography (**a**) and duplex ultrasound (**b**), both showing the occluded transverse sinus (*). The patient was later extubated, underwent cranioplasty, and recovered well. In our setting, TUS proved useful for visualizing the larger venous vessels, particularly in cases of elevated ICP, without requiring lowering the upper body for CT imaging.

During inpatient treatment, TUS was applied to address alternative clinical questions. In the first case, a 42-year-old female patient suffered a transverse sinus thrombosis with subsequent venous hemorrhage. Due to rapid deterioration of consciousness, the need for intubation, and a space-occupying hemorrhage, a decompressive hemicraniectomy was performed. With fluctuating intracranial pressure (ICP) peaks, follow-up imaging was primarily conducted using ultrasound. The occluded transverse sinus could be visualized daily (Figure 8).

**Figure 9:**
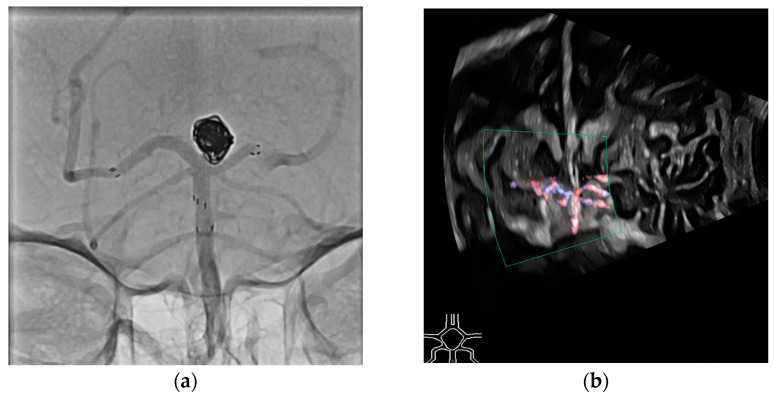
Digital subtraction angiography (**a**) and duplex ultrasound (**b**) of the basilar artery treated with a Y-stent. Clear perfusion is visible in the duplex ultrasound, and in this case, the need for follow-up CT angiography to rule out arterial occlusion was avoided. This patient also recovered well and was soon transferred to rehabilitation.

In the second case, a patient experienced a SAH due to a ruptured basilar tip aneurysm. The aneurysm was treated with coil embolization and a Y-stent. Given the early postoperative period, dual antiplatelet therapy was not feasible. Ultrasound was used to monitor the patency of the stent and to exclude in-stent thrombosis (Figure 9).

As described previously, the application of TUS, particularly as TCD for assessing intracerebral vascular flow profiles, has been well documented for over 40 years [11,43]. Although the present study focuses on the morphological assessment in the absence of cranial bone, TCD is widely recognized as a standard tool for evaluating vasospasms in many clinics following aneurysmal subarachnoid hemorrhage (SAH). Additionally, several studies have demonstrated the value of TUS in the perioperative setting, particularly after craniotomy for malignant brain tumors or arteriovenous malformations (AVMs) [15,44,45]. While digital subtraction angiography (DSA) remains the gold standard for imaging AVM formations and detecting residuals, non-invasive methods such as MRI—with advanced sequences like arterial spin labeling (ASL)—are gaining increasing attention [46]. Within the framework of non-invasive and radiation-neutral techniques, TUS could continue to play an important role in imaging hemorrhagic vascular accidents, which in our hospital (and the present study) are the prevalent ones. Its integration with MRI/ASL may potentially reduce the need for CT-A and DSA in pre- and perioperative settings, thereby refining the indications for invasive and radiation-intensive imaging procedures. 

### 4.6. Study Limitations

In addition to the limited number of patients, the major limitation of this study is the lack of standardization due to its retrospective design. Both qualitative and quantitative data were available from previously stored records. During patient treatment, focused ultrasound examinations were often conducted with specific questions in mind, without special attention to the optimal visualization of all structures relevant to this study. It can therefore be assumed that this affected the qualitative assessment by the examiners (structures that could have been well visualized but were not recorded) and the quantitative analysis (potentially better correlation with more available data). 

Although sequences of TUS and CT in between surgical interventions, re-bleeding, or changes in the CSF drainage regime were excluded from the quantitative assessment, other non-standardized factors such as patient positioning (degree of upper body elevation in TUS) during each examination may have impacted the results. Ultimately, our neurosurgical ICU usually does not treat polytrauma cases, but rather, primarily spontaneous intracranial hemorrhages/infarcts or, in some cases, isolated traumatic brain injuries without accompanying injuries. As a result, polytrauma patients are underrepresented compared with a typical patient population.

## 5. Conclusions

Transcranial ultrasound is a safe, cost-effective, and time-efficient method that may become increasingly relevant for imaging post-hemicraniectomy patients. In our experience, transcranial ultrasound was effective in visualizing intraventricular catheter placement, the ventricular system, midline and space-occupying lesions. The present study showed a strong correlation in terms of midline shift, lateral ventricular width and intracerebral hematomas between computed tomography and transcranial ultrasound. Further enhancements in image quality could potentially reduce the overall need for CT scans.

## Figures and Tables

**Table 1:** Clinical and demographic data (Abbreviations: F: female; M: male; CT: computed tomography; TUS: transcranial ultrasound).

Characteristics (Unit)	Proportion/Value
Sex (patients)	
F	63.2% (12/19)
M	36.8% (7/19)
Primary condition	
Aneurysmatic subarachnoid hemorrhage (SAH)	57.9% (11/19)
Anterior communicating artery aneurysm	36.4% (4/11)
Middle cerebral artery aneurysm	36.4% (4/11)
Pericallosal artery aneurysm	9.1% (1/11)
Posterior communicating artery aneurysm	9.1% (1/11)
Basilar artery aneurysm	9.1% (1/11)
Malignant media infarction	21.1% (4/19)
Spontaneous intracerebral hemorrhage	15.8% (3/19)
Right hemisphere	33.3% (1/3)
Left hemisphere	66.6% (2/3)
Sinus transversus thrombosis with hemorrhage	5.2% (1/19)
Mean patient stay (days ± standard deviation)	19.53 ± 10.65
Mean CT imaging per patient and stay (count ± standard deviation)	4.42 ± 2.57
Mean TUS sessions per patient and stay (count ± standard deviation)	8.32 ± 5.87

**Table 2:** Summarized mean ratings on the Likert scale and rating difference between both subgroups. (Abbreviations: EVD: external ventricular drainage; ICH: intracerebral hematoma).

Image Marker	Residents	Attending Physicians	Mean Rating Difference
Ventricular system	1.37	1.35	0.02
Midline	2.14	1.51	0.63
Subdural space	2.84	2.29	0.55
EVD location	3.21	2.28	0.93
Cortical gyration	3.21	2.46	0.75
ICH	3.12	2.68	0.44
Infarction area	4.86	4.50	0.36

**Table 3:** Comparison studies of intracranial imaging findings and correlation between computed tomography (CT) and transcranial ultrasound (TUS) in patients with or without prior craniectomy (Abbreviations: V3: third ventricle; V4: fourth ventricle; ML: midline; (r): right; (l): left; ICC: intraclass correlation coefficient; ρ: Spearman’s Rho coefficient; LVW: lateral ventricular width; m: mean).

Reference	Number of Patients	Time Frame Between CT/TUS	TUS Through Craniectomy Defect?	Image Findings and Statistical Correlation
De Bonis et al., 2020 [7]	10	30 min	Yes	LVW; ρ = 0.99 V3; ρ = 0.9 Cella media width; ρ = 0.95
Caricato et al. (2012) [30]	30	TUS immediately before CT (only first and last TUS/CT per patient included)	Yes	ML shift; ICC = 0.98 LVW; ICC = 0.97 Focal CT-hyperdense lesions (volume); ICC = 0.99 Focal CT-hypodense lesions (volume); ICC = 0.062
Chouhan et al. (2024) [24]	40	<2 h	Yes	Lateral ventricle diameter (r); ICC = 0.93 Lateral ventricle diameter (l); ICC = 0.96
Bendella et al. (2017) [6]	102	<24 h	Yes	Lateral ventricle diameter (r); r = 0.98 Lateral ventricle diameter (l); r = 0.98 V3 diameter; r = 0.99 V4 diameter; r = 0.95 ML shift; r = 0.99
Oliveira et al. (2017) [27]	15	<6 h (only first TUS/CT per patient included)	No (temporal window)	ML shift; ICC = 0.93 V3; ICC = 0.88
Seidel et al. (1996) [28]	18	<12 h	No (temporal window)	Dislocation of V3 from ML; r = 0.87
Widehem et al. (2021) [5]	100 (87 successfully included)	<1 h	No (temporal window)	V3 diameter ICC (right window) = 0.90 ICC (left window) = 0.92
Lasselin et al. (2022) [29]	177	87 (m) ± 73 min	No (temporal window)	ML shift; r^2^ = 0.96 V3 diameter; r^2^ = 0.96

## Data Availability

The authors will make the raw data supporting this article’s conclusion available upon request.
